# Naturally Occurring Compounds Elicit HIV-1 Replication in Chronically Infected Promonocytic Cells

**DOI:** 10.1155/2014/989101

**Published:** 2014-05-12

**Authors:** Andrea Alejandra Barquero, María Eugenia Dávola, Diego Ariel Riva, Susana Esther Mersich, Laura Edith Alché

**Affiliations:** Laboratory of Virology, Department of Biological Chemistry, IQUIBICEN, School of Science, University of Buenos Aires, Ciudad Universitaria, Pabellón 2, 4to. Piso, Intendente Güiraldes 2160, C1428EGA Buenos Aires, Argentina

## Abstract

Since antiretroviral therapy suppresses but does not eradicate HIV-1 infection, methods to purge viral reservoirs are required. Many strategies involve the reactivation of chronically HIV infected cells to induce the expression of integrated viral genome. In this study, five bioactive compounds, the plant derivatives 1-cinnamoyl-3,11-dihydroxymeliacarpin (CDM), nordihydroguaiaretic acid (NDGA), and curcumin (Cur) and the synthetic stigmasterol analogs (22S,23S)-22,23-dihydroxystigmast-4-en-3-one (compound **1**) and (22S,23S)-3**β**-bromo-5**α**,22,23-trihydroxystigmastan-6-one (compound **2**), were evaluated for their ability to elicit HIV replication in promonocytic (U1) and lymphocytic (H9+) HIV-1 chronically infected cells. The results revealed that natural compounds CDM, NDGA, and Cur were able to increase HIV-1 p24 antigen, determined by ELISA, only in latently infected promonocytic cells. CDM would reactivate HIV from latency by modulating the release of IL-6 and TNF-**α**, since the amount of both cytokines measured through ELISA significantly increased in U1 treated cells. Besides, NDGA increased ROS production, which might be related to the increase on p24 level observed in NDGA treated U1. These findings suggest that CDM, NDGA, and Cur might be candidates for further studies on latency-reversing therapeutics to eliminate latently HIV-1 reservoirs.

## 1. Introduction

The highly active antiretroviral therapy (HAART) effectively suppresses human immunodeficiency virus replication (HIV) but does not target provirus from chronically infected cells. Therefore, interruption of treatment results in a rapid viral rebound arising from infected cell reservoirs such as macrophages and resting memory CD4+ T-cells or sanctuary sites, where drug penetration is suboptimal [1]. One current strategy to HIV eradication is the use of molecules that specifically induce the expression of the integrated HIV genome, making latently infected cells vulnerable to immune-mediated killing, plus antiretroviral therapy to block new infections [1]. In this sense, a number of approaches have been studied for the reactivation of latently HIV-infected cells, including cytokines such us IL-2 and IL-7 or small molecules with pharmacological properties that allow them to access the viral reservoirs [2, 3].

The limonoid 1-cinnamoyl-3,11-dihydroxymeliacarpin (CDM) and the polyphenolic compounds nordihydroguaiaretic acid (NDGA) and curcumin (Cur) are plant constituents with several biological activities. CDM, isolated from leaves extracts of* Melia azedarach* L. [4], displays immunomodulatory and antiviral properties [5, 6]. The bioactive compound NDGA isolated from creosote bush presents anticancer, immunosuppressive, antioxidant, antimicrobial, and anti-inflammatory activities and is potentially useful in treating different diseases [7]. Cur, a component of turmeric spice, also has therapeutic significance in cancers, arthritis, allergies, neurodegenerative disease, hepatic disorders, and autoimmune diseases [8]. On the other hand, the synthetic steroids structurally related to brassinosteroids, plant growth hormones, named compound** 1** ((22S,23S)-22,23-dihydroxystigmast-4-en-3-one) and compound** 2** ((22S,23S)-3*β*-bromo-5*α*,22,23-trihydroxystigmastan-6-one), have antiviral and immunomodulatory activities [9–11].

The fact that these natural and synthetic compounds exhibit diverse bioactivities strongly suggests that they may have various targets, some of which have been already described [7, 12]. Since HIV reactivation may be accomplished at the molecular level through multiple mechanisms, our experimental work has been focused to investigate whether natural CDM, NDGA, and Cur and synthetic** 1** and** 2** compounds can elicit HIV replication from U1 and H9+ cells, two chronically infected monocytic and lymphocytic cell lines, respectively.

## 2. Materials and Methods

### 2.1. Cells and Compounds

Human lymphocytic H9 and promonocytic U937 (ATCC) uninfected cell lines and their respective HIV-1 chronically infected cell lines H9/HTLVIIIB (infected with HIVHXB2 strain, designated H9+) and U1 (subclone of U937 cells that contain two integrated copies of HIV-1 proviral DNA) were provided by the NIH AIDS Research and References Reagent Program. Cells were cultured with RPMI 1640 medium supplemented with 2 mM L-glutamine, 100 *μ*g/mL streptomycin, 100 IU/mL penicillin, and 10% fetal bovine serum (FBS) at 37°C in a humidified atmosphere (5% CO_2_ in air). Cells were collected during the log phase of growth when cell viability was over 95% and immediately used for the experiments described below.

CDM was purified from leaves of* M. azedarach* L., as described by Alché et al. [4], solubilized in RPMI 1640 to a final concentration of 1.5 mM. NDGA provided by Dr. B. Koningheim, Universidad Nacional de Córdoba, Argentina, was dissolved in DMSO at 60 mM and diluted with RPMI 1640 medium. Cur (Sigma Chemical Co. St. Louis, MO) was dissolved in DMSO at 10 mM and diluted with RPMI 1640 medium. Compounds** 1** and** 2** were synthesized as described by Michelini et al. [9, 11], dissolved in DMSO at 10 mM, and diluted with culture medium for testing.

### 2.2. Cell Viability

Cell viability was determined by the Trypan blue dye exclusion assay. A suspension of 1–5 × 10^5^ cells/mL was placed in 24-well plates. After treatment with several concentrations of each compound in triplicate for 48 h, cells were stained with 0.4% Trypan blue for 5 min at room temperature and counted in a Neubauer cell chamber. The total number of viable cells in the control group was considered as 100% viability and the concentration of each compound required to reduce cell viability by 50% (CC_50_) was calculated. Control with 1% DMSO showed no effect on cell viability.

### 2.3. Virus Production Assay

Cells were treated with several concentrations of each compound (in triplicate) for 48 h and, then, HIV-1 production was measured through the quantification of viral p24 antigen concentration in U1 and H9+ cell supernatants by an enzyme-linked immunosorbent assay (ELISA) (Vironostika, Biomerieux), according to the manufacturer's instructions. Control with 1% DMSO showed no effect on p24 antigen concentration.

### 2.4. Cytokine Determination

Human TNF-*α* and IL-6 cytokines accumulated in cell supernatants were quantified by commercial ELISA sets (BDOptEIATM, Becton Dickinson, USA) according to manufacturer's instructions, and results were expressed as percentage with respect to untreated control cells.

### 2.5. Reactive Oxygen Species Production

U1 cell line was incubated in the absence or presence of NDGA or Cur for 48 h. Then, to detect intracellular production of reactive oxygen species (ROS), incubations with 50 *μ*M oxidation-sensitive dichlorodihydrofluorescein diacetate (DCFH-DA, Sigma) for 30 min at 37°C in the dark were done. Cells were washed twice with cold PBS, suspended in PBS at 5 × 10^5^cells/mL, and lysed with 1% Triton X-100 and the fluorescence intensity was then monitored with a fluorescence spectrophotometer (Shimadzu RF-510, excitation wavelength 485 nm, emission wavelength 530 nm). Fluorescence intensity of untreated control cells was set as 1 and results were expressed in X-fold.

### 2.6. Statistical Analysis

All data represent means ± SD from three separate experiments. Student's* t*-test was used for statistical analysis of all data with *P* = 0.05.

## 3. Results

### 3.1. Effect of the Compounds on HIV-1 Production in Chronically Infected Cells

Initially, the cytotoxicity of natural compounds (CDM, NDGA, and Cur) and synthetic stigmasterol analogs** 1** and** 2** on uninfected and HIV-1 chronically infected cells was evaluated by Trypan blue dye exclusion method.

**Figure 1:**
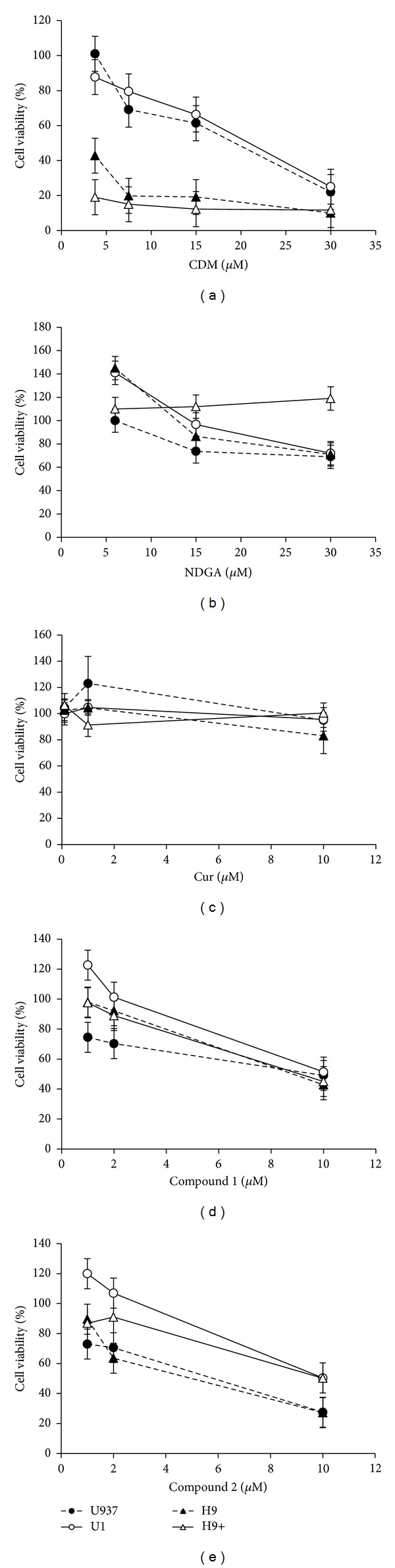
Dose-dependent effect of natural and synthetic compounds on uninfected and HIV-1 chronically infected cell viability. Promonocytic (U937 and U1) and lymphocytic cells (H9 and H9+) were treated with different concentrations of each compound for 48 h and cell viability was determined by measuring the exclusion of Trypan blue. Results were expressed as the percentage of total number of viable cells in the presence of the tested compound with respect to untreated cells. Data represent means ± SD from three separate experiments.

As shown in Figure 1(a), H9 and H9+ cells were very sensitive to CDM in comparison to promonocytic cells since CC_50_ values obtained were <3.75 *μ*M for lymphocytic cells and 19.5 *μ*M and 21 *μ*M for U937 and U1 cells, respectively. NDGA does not seem to affect cell viability of any of the four cell lines assayed at concentrations up to 30 *μ*M while Cur showed no significant cytotoxic effect on noninfected and HIV-infected cells at concentrations up to 10 *μ*M (Figures 1(b) and 1(c), resp.). When synthetic analog** 1** was assayed, CC_50_ values obtained were 10 *μ*M for U937 and U1 monocytic cells and 8.8 *μ*M for H9 and H9+ lymphocytic cells (Figure 1(d)). The concentration of compound** 2** required to produce 50% cytotoxicity was estimated to be 10 *μ*M for HIV-infected cells U1 and H9+ and slightly lower for noninfected cells (6 *μ*M for U937 and 5 *μ*M for H9) (Figure 1(e)).

**Figure 2:**
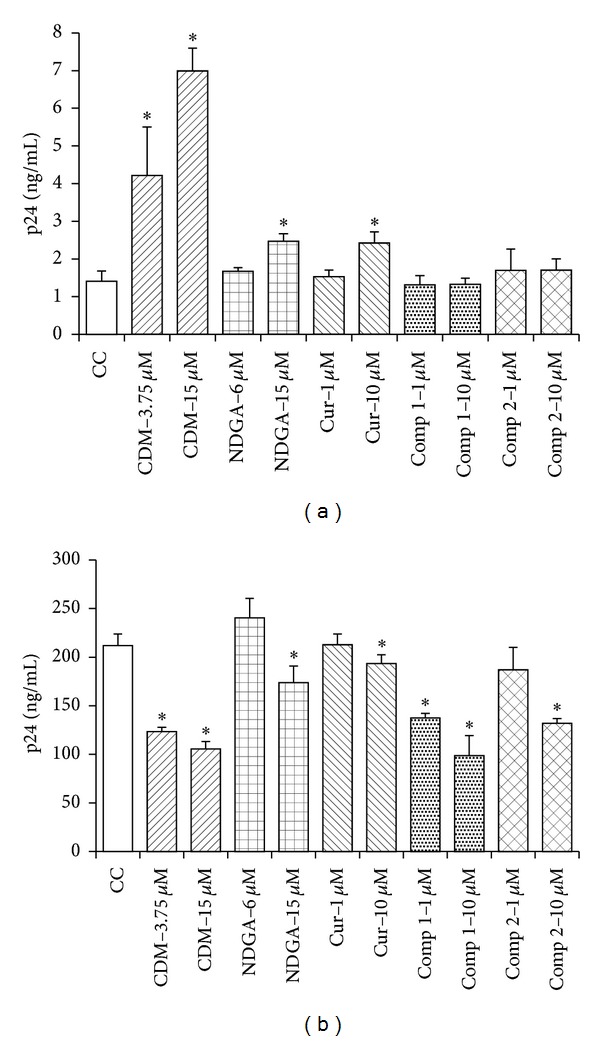
Effect of natural and synthetic compounds on p24 production in HIV-1 chronically infected cells. Monocytic U1 (a) and lymphocytic H9+ (b) cells were treated with different concentrations of CDM, NDGA, and Cur and compounds** 1** and** 2**, during 48 h, in triplicate. p24 antigen was determined in cell supernatants by ELISA. Values and bars are means ± SD from three separate experiments.  *P = 0.05, significantly different from untreated control cells (CC).

Then, we analyzed the ability of CDM, NDGA, and Cur and synthetic compounds** 1** and** 2** to alter the production of HIV from U1 and H9+ chronically infected cells. Of the five tested compounds, CDM, NDGA, and Cur showed a dose-dependent activation of HIV-1 replication in U1 cells, whereas no increase in p24 levels was observed in U1 cells when synthetic analogs** 1** and** 2** were added (Figure 2(a)). At the highest concentration tested, CDM increased p24 production by approximately sevenfold and both NDGA and Cur by nearly twofold compared with untreated control cells (Figure 2(a)).

Conversely, H9+ cells treated with the highest concentration of any compound rendered significant lower levels of p24 antigen with respect to untreated control cells (Figure 2(b)).

Therefore, natural compounds CDM, NDGA, and Cur were able to activate HIV-1 replication only in latently infected promonocytic cells.

### 3.2. Cytokine Secretion in HIV-1 Chronically Infected Promonocytic Cells Treated with CDM

Proinflammatory cytokines TNF-*α*, IL-2, IL-6, and IL-7 have been shown to upregulate the expression of HIV-1 [13–15]. Since CDM modulates TNF-*α* and IL-6 secretion in stimulated macrophages [6], its effect on cytokine production in HIV-1 chronically infected macrophages has been investigated. Thus, U1 cells were treated with 15 *μ*M CDM for 48 h and, then, IL-6 and TNF-*α* levels accumulated in the supernatants were measured by ELISA. It was found that CDM increased the amount of both IL-6 and TNF-*α* since cytokine levels were significantly higher in U1 treated macrophages than in control cells (477% and 170%, resp.) (Table 1).

Therefore, CDM also exerted an immunomodulatory effect on HIV-1 persistently infected promonocytic cells.

### 3.3. ROS Production in HIV-1 Chronically Infected Promonocytic Cells Treated with NDGA and Cur

It has been proposed that ROS can indirectly activate latent HIV-1 [16]. Considering that NDGA and Cur are known to affect intracellular redox state, we hypothesized that the induction of an oxidative stress by both drugs might play a role in the reactivation of latent virus. To test this assumption, U1 cells were treated with different concentrations of NDGA and Cur for 48 h and, then, processed to quantify intracellular ROS using the oxidation sensitive dye DCFA-DA. Fluorescence intensity of untreated cells was set as 1, and results were expressed as X-fold. As shown in Table 2, 15 *μ*M NDGA significantly increased ROS production in U1 cells to 140% compared with untreated control cells. The treatment with 1 or 10 *μ*M Cur showed no effect on the redox state in this cellular system (Table 2).

These results suggest that HIV-1 reactivation in NDGA treated U1 cells might be ascribed to the modulation of oxidative conditions.

## 4. Discussion

The existence of viral reservoirs that harbor latent forms of HIV hinders the success of HAART. Activation of latent proviruses from infected cells in combination with HAART that prevents the spread of free virus to adjacent cells is part of a therapeutic strategy which is evaluated in chronically infected cell line systems [17].

In the present study, natural compounds such as CDM, Cur, and NDGA proved to induce viral replication only in latently HIV-1-infected promonocytic cells (Figure 2).

The response of latently infected U1 cells to treatment with CDM, NDGA, and Cur was similar to that reported with other natural compounds such us 10 *μ*g/mL* Ephedrae herba* and 50 *μ*M Resveratrol, which increased HIV- p24 levels by five-sevenfold in chronically infected cells [18, 19].

In the case of H9+ lymphocytic cells, the decrease in p24 levels obtained with CDM and compounds** 1** and** 2** may be ascribed to a reduced number of viable cells (Figures 1(a), 1(d), and 1(e)). In a previous report, the reduction in p24 levels in H9+ cells treated with 10 *μ*M Cur alone or in combination with the protease inhibitor indinavir was also observed [20]. Besides, NDGA and its derivatives have been found to inhibit HIV-Tat regulated transactivation in human epithelial cells [21].

This dual response observed in p24 yield depending on the source of HIV chronically infected cells has been already described in other experimental models and can be attributed not only to the different lineage (macrophages and lymphocytes) but also to the type of chronic infection established in these cells, latency in U1 and persistence in H9+ [22].

Considering the complexity of HIV reactivation, it will require much additional work to understand the mechanisms responsible for p24 antigen induction by CDM, NDGA, and Cur, but a few potential explanations may be considered. In the case of CDM, we previously reported that CDM decreases IL-6 production in HSV-1-infected corneal cells, enhances TNF-*α*, and reduces IL-6 secretion when macrophages are either infected with HSV-1 or stimulated with LPS [6]. Instead, herein CDM was able to induce not only TNF-*α* production but also IL-6 release from U1 cells (Table 1). This cell line is one of the most exhaustively described models of postintegration latency, characterized by low levels of constitutive virus expression that can be modulated by cytokines, such as IL-6 and TNF-*α* [14, 18, 23]. Therefore, we conclude that CDM reactivated HIV from latency by modulating the release of IL-6 and TNF-*α* in HIV-1 latently infected U1 cells.

On the other hand, the literature reported that NDGA and Cur may possess antioxidant effects as well as prooxidant activity, dependent on dose and chemical environment [7, 24, 25]. It is well established that, depending on the level of ROS, different transcription factors are activated and produce distinct biological responses [26]. Then, the oxidative effect of NDGA on U1 treated cells might be linked to the increase in p24 level. In contrast, since Cur activates HIV expression without enhancing ROS levels in U1 cells, the purge of latency would not be associated with a dysfunction of cellular redox control (Table 2). Based on Cur ability to affect multiple targets, other signaling pathways might be involved.

Although the exact intracellular mechanisms of these natural compounds are still not fully understood our findings might be considered a relevant contribution to design efficient therapeutic strategies for attacking latent HIV reservoirs.

## Figures and Tables

**Table 1:** Effect of CDM on cytokine secretion in HIV-1 latently infected U1 cells.

Treatment	IL-6 (pg/mL)	TNF-*α* (ng/mL)
Control cells	44.65 ± 3.7	1.65 ± 0.44
CDM (15 *μ*M)	213.07 ± 17	2.81 ± 0.77

**Table 2:** Effect of NDGA and Cur on ROS production in HIV-1 latently infected U1 cells.

Treatment	DCFH-DA (X-fold)
NDGA (6 *μ*M)	1.00 ± 0.10
NDGA (15 *μ*M)	1.40 ± 0.10
Cur (1 *μ*M)	1.01 ± 0.22
Cur (10 *μ*M)	0.91 ± 0.27
